# Supplementary material for: Complex‐centric proteome profiling by SEC‐SWATH‐MS
Source: Mol Syst Biol. 2019 Jan 14;15(1):e8438. doi: 10.15252/msb.20188438 (PMC6346213; doi:10.15252/msb.20188438)
Supplement: Supplementary file 8 — Dataset EV7 [file MSB-15-e8438-s008.zip › feature_plots_string/P11498.pdf]

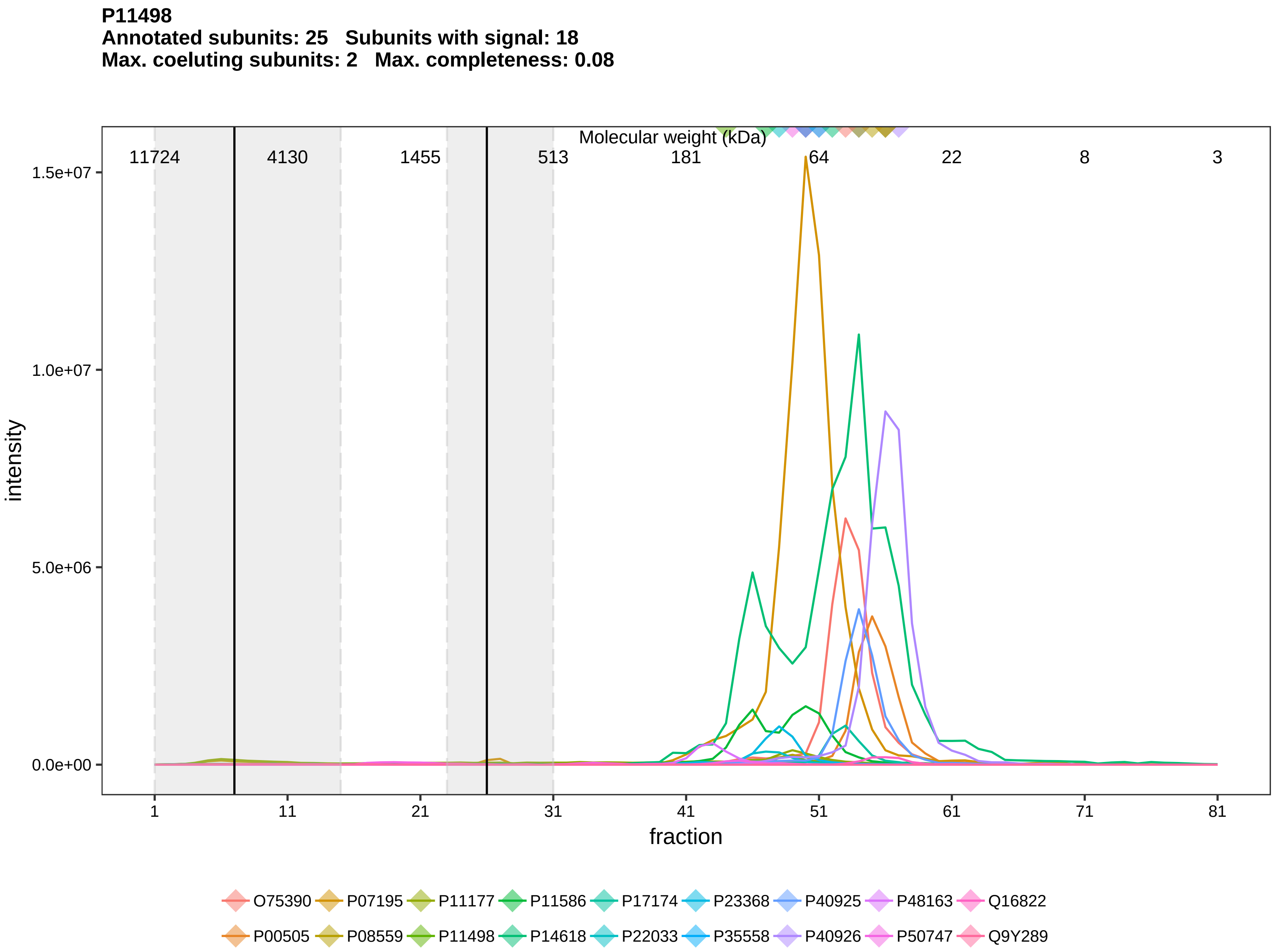

P11498
Annotated subunits: 25 Subunits with signal: 18
Max. coeluting subunits: 2 Max. completeness: 0.08
Molecular weight (kDa)
11724
4130
1455
513
181
64
22
8
3
intensity
1.5e+07
1.0e+07
5.0e+06
0.0e+00
fraction
1
11
21
31
41
51
61
71
81
O75390
P07195
P11177
P11586
P17174
P23368
P40925
P48163
Q16822
P00505
P08559
P11498
P14618
P22033
P35558
P40926
P50747
Q9Y289
